# Observations on Dr. Moseley's Case of Hydrophobia

**Published:** 1808-03

**Authors:** 


### *To the Editors of the Medical and Physical Journal.*

GENTLEMEN,

IN the Sun newspaper of January 26, 1808, I read over a Case of Hydrophobia successfully treated by Dr. Moseley; the singularity of the event, and the confidence with which he asserts its rabid reality, has given rise to the following observations, which, should they appear worthy insertion in your valuable miscellany, it would give considerable pleasure to

Your most obedient Servant,

J. D.

---

NOTHING is more dangerous to society, and destructive to the comfort of invalids, than the exhibition of a powerful medicine; even in slight cases of disease in the hands of uninformed and unprofessional beings. But when a disorder has arisen, whose termination, even when treated by the scientific practitioner, is always in death; and at the same time, when promptly attended to, every bad consequence can be obviated, how much more ought we to lessen the confidence of the public in any remedy, and rather than recommend one, exhort by all means their immediate application to the skilful practitioner. Why Dr. Moseley should advertise in a newspaper, perused by almost every body but those of the profession, the good effects of *mercury* in Rabies Canina is inexplicable. However, whatsoever might have been the motive which led the Doctor to its publication, it ought, certainly, to be investigated with that caution and criticism which every philosophic and judicious practitioner will readily submit to.

Dr. M. has of late bore a very conspicuous character; his genius is confessed by the most inveterate of his enemies, but his accuracy, I am sorry to say, has too frequently proved to be of so ambiguous a nature, as to perplex and embarrass every principle of science and ratiocination. His observations, so flattering to humanity, and so confidently asserted, (that had we not known the man, his every word would appear sanctioned with undeniable facts) are found to be nothing but the wild effusions of a jealous and ambitious spirit. His cases, arguments, and insinuations on vaccination, have so evidently displayed his fallacy, as to justify this opinion. The calm dispassionate reasonings of Rowland Hill, and the indefatigable examinations of the philanthropic Ring, are too public to merit any remarks from me on the rashness of the Doctor. His influence, however, on the public mind, is still prevalent; still does he publish placards abounding in every species of delusion, and still offer the most gross misrepresentations in every popular work an Englishman looks into. Thus, without that integrity, which makes a man believed by the most profligate, how are we to judge of the truth of the case he has so peremptorily alleged. In the first place, to whom has he addressed this singular interposition of fortune; surely not to the Faculty, for if so, it would have appeared in a medical work; no, he then would have been regarded as he deserved; but, according to his usual practice, he has imposed it on the public, who, as ignorant of philosophy, as biased by predilection, will receive it with all the avidity and thankfulness that could be wished for. If he meant to enlighten them, why not first subject it to the analysis of men, eminent for their professional capacity? why not ascertain the certainty of its hydrophobic tendency by pathologists venerated for their honour and discernment? indeed, I know not, without his views were (as customary) to prejudice the general mind in his favour, both as to his experience and his abilities; and by bringing it thus so completely under his power, his interested attempts tempts of exterminating the divine gift of vaccination from the kingdom may be effected, and thus accomplish, if not a fortnne, his heart's desire.

I shall confine myself more strictly to the subject of his communication, and should there be any inaccuracy in my comments, it has neither originated from malice or revenge, but literally from an error in judgment. I am firmly convinced, that the approaching disease partook more of the characteristic features of Tetanus than genuine Hydrophobia. Whether I consider the cause, consequence, treatment, or success, all will tend to corroborate it. No place in the human system is more replete with nerves than the termination of the fingers, hence the acuteness of feeling in those parts so invariably manifested by their employment as our instruments of touch, and on the same reason the utility of the nails to preserve them from the continued routine of accidents they are daily exposed to. Dr. Moseley informs us, " that two punctures were made near the end of the little finger, which, though small, must have been very deep, as the patient says the pain in her finger for two hours was excessive. When the severity of the pain abated, a sensation came on like the pricking of pins, which continued for about a quarter of an hour, and then *ceased.* "On the 4th and following days it returned, and on the 11th she was attacked with numbness of the finger and hand." How far these punctures might have been conducive to Tetanus we could easily explain, had the habit of the patient been noticed; but this the Doctor has very ingeniously omitted.

The sleepy and settled appearance of the dog, which in contradistinction to the general phenomena of Rabies, the canine race are marked by a peculiar wandering and restless disposition, may weigh somewhat against the inference of the true hydrophobic character of the dog. Dr. Hamilton says, we discover some strange dog affected with this complaint communicating it as he passed through towns and villages; and in carrying this research a degree farther, we may be able to find the same dog the property of some person in a distant country, for a dog may *wander* to a great distance while under this disease, after his knowledge of home and his former connections have deserted him. However, it must be confessed, that torpor and stupidity are very prevailing symptoms.

The quick accession of the disease, being only eleven days after the bite, is a circumstance unprecedented in any case of rabid hydrophobia, substantiated by men of truth,

 science, science, and experience. From the celebrated Dr. Fothergill, we learn that the most usual time before the disease breaks out, is from three to eight weeks; but in the present case, the disorder was in every respect abated on the seventeenth day, for the subsequent derangement and sickness may be; indeed, he himself has attributed it to the effects of the mercury.

In Dr. Bardsley's able remarks on hydrophobia, we have not only his, but the authority of many highly acquainted with, and who have paid indefatigable attention to the subject, that hydrophobia generally appears between a month or six weeks after the bite. How then are we to judge? Shall we refer to the symptoms Mrs. Lacase laboured under? Alas! I am fearful they will afford still more negative conclusions. What did the severity of the pain, the occasional numbness of the finger, and contraction of the muscles of the hand denote more evidently than the partial division or lesion of a nerve. Again, "the oppression of the chest" is a certain concomitant on Tetanus. Her dislike to water appears the grand fabric on which Dr. Moseley has built such positive deductions. For, "in the morning, as she was drawing some water from the cistern in the yard, she was seized with a trembling and giddiness of head, and terror at the sight and *noise* of the water running into the pail." It appears to me, "from the whole of her system being extremely affected, and her head so distracted with *noise*, like the running of coaches," was enough to make her uneasy, independent of the sound from the impetuosity with which water generally flows. However, to confirm his suspicion, "of the mischief at hand," the Doctor brought a "pewter bason filled with water, and slopped it about before her; but on much agitating it, and pouring it out of the bason into a pewter pot, and from thence, back into the bason a few times, she looked at it with horror, and was so distressed, without having any idea of his motives, that she begged he would take it away." In my opinion, bringing water in the manner he did, agitating it in the two vessels, with a countenance anticipating some "dreadful event from woeful experience," together with keeping his intentions clandestinely from the patient, might alone alarm her. In all probability she was in the greatest dread of his going to dash it over her, or at least, of trying some experiment with her; the idea of which I amply know will terrify the general race of beings more than almost any thing. She could bear no more the light of a candle than the bason of water; her eyes were so so sensible from the irritability of the nervous system, that any other dazzling body would have produced the same effect. Myself, who do not at all pretend to be of the nervous temperament, (how much more so those who are) will travel miles in the shade without any ailment; but should the sun shine, and the ground be covered with snow, or should I be walking next the sea, the consequence is a pain and giddiness in the head, symptoms though in a far inferior degree, precisely the same as those complained of in the case of Hannah Lacase, after the application of a brilliant body to the eyes.

The never failing companion, the most terrible, astonishing, and unfortunate symptom, was here completely absent; she could swallow liquids without the least difficulty; no convulsions excited, no spasms augmented so melancholy in the hydrophobic sufferer. Indeed, she took a draught of more than two ounces every four hours without any complaint either as to her beholding or swallowing it; on this she could look without any dread; and why? she had knowledge of its purpose, and confidence of its success. Before I refer to the treatment, the success of which, perhaps, depended greatly on the sudorifics employed, I shall briefly lay down a few select cases of the similarity of instances of tetanus and hysteria to hydrophobia, as cited by authors and practitioners, whose talents, while they shed knowledge and skill on the aspiring individual, will so far sanction what he has learnt, that he may practice in direct subserviency to his instructions, confident that he has gained not only information, but met with solid and undeniable truth. The frequent occurrence of an aversion to fluids, and of great difficulty in swallowing them, says the candid Dr. Bardsley, (vide p. 279 Med. Reports) in women affected with hysteria, has been noticed by many writers. Some of these facts demonstrate that all the spmptoms of canine madness have been brought on by violent affections of the mind in irritable and delicate habits. Dr. B. here quotes a case from Platerus, of a singular instance of hydrophobia in consequence of *terror.* A woman of an irritable state of nerves, was much alarmed at being left alone by her companions on the banks of a river, where she had been employed in washing linen. As the evening approached, her fears increased. After returning home, she was seized with a violent sobbing, and was almost in danger of suffocation. These symptoms increased daily, and an utter aversion to fluids supervened. The motion of the air and the appearance of luminous objects were equally

 offensive.

offensive. She expired under the pressure of these symptoms on the eighth day of the disease. He mentions another instance from Sauvages, of a fatal example of hydrophobia in a young woman, in consequence of agitation of the mind during a morbid and irritable state of the body. Mr. Wilmer of Coventry, has related a circumstance of striking similitude between tetanus and hydrophobia. The young gentleman had the middle finger slightly wounded by a splinter of wood. In the course of twelve days he complained of stiffness of the throat and neck; a pain was felt about the scrobiculus cordis, watery drinks were found impossible to be swallowed, and whenever they approached his mouth, the convulsive spasms returned, and mucus collected plentifully in the corner of the mouth. Drs. Rush and Percival also strongly corroborate the occurrence of hydrophobia in tetanus, where no rabid virus could ever have been absorbed.

Although I have been endeavouring to elucidate the error Dr. Moseley has led himself into, by considering the state of Hannah Lacase to have arisen from the absorption of hydrophobic virus, I strenuously recommend and ardently extol the practice he so efficaciously pursued, and readily communicated, though it certainly is ridiculous folly to have inserted such in a public newspaper. The promulgation of one fact, however contrary to the garb it is arrayed in, will tend, like the sowing of seed, to the production of more.

Dr. M. removed the skin where the wounds had been, and instituted a drain, which was continued during the whole treatment. An ounce of ungt. mercuriale fortius was rubbed in, twice in twenty-four hours, and the following draught ordered to be taken every four hours, with directions to promote perspiration every way possible.

℞. Julep e camphor. unc. ij. Spt. volat. aromat. Rad. valer. pulv. aa. dr. ½. M. et fiat haustus.

We hear by the next day, her gums were sore by the frictions; great perspiration, and every calamity abated.—The question now remains, to which of these three remedies, we may attribute this sudden and unexpected success. The first, or that of detaching the diseased skin, and instituting a drain, has been proved to be of singular avail in tetanus. Mr. Hicks's cases, ("Med. and Phys. Journal, p. 272, No. 17") evidently, beyond a doubt, certify it. 2dly. The sudorifics and antispasmodics here made use of, must have been remarkably beneficial, and above all, testify my opinion of its being of a tetanic nature. In all spasms and rigidity of the muscles they are our constant resources.

resources. But it may be objected, that there were no spasms; by the same reason may be opposed the existence of hydrophobia; but still, as Dr. M. says, they were "near at hand." Then, by the speedy and prompt administration of every remedy tending to relax the surface, to give ease and pliability to the muscular, and overcome the morbid irritability of the nervous system, were not these consequences so truly dreadful and dejecting, prevented. In my own practice, I have seen the most decided advantage resulting from this practice in a case of tetanus. In the course of last spring, a mixture was prescribed to the above patient as follows:

℞. Spt. amm. comp. ʒiij. Spt. lav. c. ʒij. Applicat. emp. canth. stom. Capiat haust sudor hora somni. Mistur. camph. ℥vj. Sumat coch. iij. 2a quaque hora.

The patient found almost immediate relief as soon as perspiration came on; but for the first two days, if he neglected taking his medicine, the spasms were sure to return; he was completely cured by the subsequent exhibition of calomel. 3dly. Mercury has been found in almost every instance of tetanus, the most efficacious mode we could possibly pursue; scarce an obstinate case has been treated without it in some form or other; every day are we reading of its success, and in almost every patient, particularly in this kingdom, have its predominant virtues been detected. Not a nation in the globe, enlightened in philosophy or physic, but what have added some testimony of its powerful influence in these affections; however, in this, as in every other remedy, we cannot place any implicit reliance. The propriety of this remedy in tetanus, added to its inefficacy in almost every species of hydrophobia, is almost enough of itself to authorize my assertions. I well know the assumption of an anonymous individual is not sufficient to carry such conviction as is requisite; and from this I have cited the observations of medical men, honoured for their fidelity, and revered for their understanding; therefore, should the extracts I have made, prove a tax upon the patience of my readers, or too prolix for your valuable pages, it has arisen from my love of truth that I entered into such a tedious detail.

